# Association of *HSPA1B* Polymorphisms with Paranoid Schizophrenia in a Polish Population

**DOI:** 10.1007/s12017-019-08575-1

**Published:** 2019-10-23

**Authors:** Malgorzata Kowalczyk, Krzysztof Kucia, Aleksander Owczarek, Renata Suchanek-Raif, Wojciech Merk, Anna Fila-Danilow, Monika Paul-Samojedny, Piotr Choreza, Jan Kowalski

**Affiliations:** 1 Department of Medical Genetics, School of Pharmacy with the Division of Laboratory Medicine, Medical University of Silesia, Jednosci 8, 41-200 Sosnowiec, Poland; 2 Department of Psychiatry and Psychotherapy, School of Medicine, Medical University of Silesia, Katowice, Ziolowa 45, 40-635 Katowice, Poland; 3 Division of Statistics, Department of Instrumental Analysis, School of Pharmacy with the Division of Laboratory Medicine, Medical University of Silesia, Ostrogorska 30, 41-200 Sosnowiec, Poland

**Keywords:** Genetic association, Paranoid schizophrenia, *HSPA1B*, Polymorphism, Haplotype, Suicide

## Abstract

This study aimed to find the potential association between *HSPA1B* polymorphisms and risk of paranoid schizophrenia, clinical variables of the disease, and suicidal behavior. A total of 901 unrelated Polish subjects of Caucasian origin (377 schizophrenia patients and 524 controls) were recruited. Four single-nucleotide polymorphisms (SNP) were genotyped using PCR–RFLP (rs539689, rs9281590) and TaqMan assays (rs263979, rs6547452). A strong tendency towards statistical significance (*p* = 0.051) was observed in rs539689 allele distribution between patients and controls in overall study subjects. After stratification according to gender, we found that rs539689 was significantly associated with schizophrenia in males, but not in females. The minor allele C had a protective effect in males [OR 0.73 (95% CI 0.61–0.88, *p* < 0.05)]. In addition, two SNPs (rs539689, rs9281590) were significantly associated with PANSS scores. Another important finding was a strong significant association between the *HSPA1B* rs539689 polymorphism and attempted suicide in schizophrenic patients. The C/C genotype and C allele were protective against suicidal behavior in entire sample (*p* < 0.001), in males (*p *< 001), and in females (*p* < 0.05), although associations were weaker than in males. Our findings support that *HSPA1B* gene may be involved in susceptibility to schizophrenia and clinical presentation of the disease in a sex-dependent manner, and may play a role in suicidal behavior in the Polish population of schizophrenic patients. Further independent analyses in different populations should be performed to clarify the role of *HSPA1B* in the pathogenesis of schizophrenia.

## Introduction

Schizophrenia is a severe psychiatric disorder, characterized by hallucinations, delusions, and cognitive deficits, and it affects about 1% of the population worldwide (Rees et al. 2015). Over the years, several aetiopathological theories for schizophrenia have been proposed, including neurodevelopmental (Fatemi and Folsom 2009), immunological (Müller and Schwarz 2010), and neurotransmitter-based hypotheses (Howes and Kapur 2009). High heritability estimates for schizophrenia (more than 80%) points to a major role for inherited genetic variants in its aetiology (Schizophrenia Working Group of the Psychiatric Genomics Consortium 2014). It is now established that the genetic risk is conferred by common alleles of small effect or rare alleles of large effect, distributed across different genes (Rees et al. 2015). Unfortunately, candidate gene association studies have often raised opposite findings, partly due to heterogeneity of the disorder and differences across populations. Hence, there is still a lack of fully replicated genetic susceptibility factors (Gejman et al. 2010).

Heat shock proteins (HSPs) are a family of highly conserved proteins constitutively expressed or induced in response to various stressors. As molecular chaperones, they participate in protein synthesis, folding, and transport, and after stress exposure, they prevent protein misfolding and aggregation (Benarroch 2011). It is known that HSPs are involved in various processes during development of the central nervous system (CNS), and determines the delicate balance between neuronal survival/differentiation or death (Reed-Herbert et al. 2006).

Genes encoding some of the HSP family members may be involved in the pathophysiology of schizophrenia, particularly with regards to the neurodevelopmental hypothesis, because these proteins have neuroprotective effects in the CNS due to their anti-apoptotic and chaperoning activities (Brown 2007). HSP70 family members seem to play a pivotal role in protection against CNS damage during critical stages of development (i.e., rapid cell proliferation, migration, and differentiation). In the presence of hyperthermia, ischemia, oxidative stress, and variety of other stressors, expression of HSP70 (especially heat-inducible HSP70-1a, -1b) is highly up-regulated in multiple areas of the brain, and protects against a variety of embryonic insults. Therefore, it was hypothesized that aberrant expression of HSP70 may be linked to structural brain abnormalities observed in schizophrenic patients (Bates et al. 1996).

Transgenic animal models and pre-conditioning experiments have proved that HSP70 overexpression in neurons and glia leads to neuroprotection against ischemic damage (Terao et al. 2009), and against excitotoxicity mediated by kainic acid and glutamate (Tsuchiya et al. 2003). HSP70 proteins have been revealed as powerful suppressors of neurodegeneration in animal models of neurodegenerative diseases (reviewed in Benarroch 2011). Selective overexpression of HSP70 has also been found to play a role in synaptic protective mechanisms that preserve neurotransmission processes during time of stress. The presence of HSP70 at the synapse following hyperthermia could facilitate the repair of stress-induced damage to synaptic proteins (Bechtold et al. 2000). Convergent findings from various areas of investigation have suggested that synaptic dysfunction results in aberrant neuronal connectivity might be the core feature of schizophrenia.

There have been reports of an elevated serum level of antibodies against HSP70 in patients with schizophrenia (Schwarz et al. 1999; Kim et al. 2001). Since HSPs are involved in various neuroprotective mechanisms, immunoreactivity against them may inhibit neuroprotective functions (Kim et al. 2008).

Three genes (*HSPA1A*, *HSPA1B*, and *HSPA1L*) encoding proteins that belong to the HSP70 family are mapped to the major histocompatibility complex (MHC) class III region on chromosome 6p21.3 (Milner and Campbell 1990). Multiple genome wide association studies (GWAS) revealed a possible involvement of the MHC region in schizophrenia susceptibility (Bergen et al. 2012; Jia et al. 2012). The latest combined analysis of GWAS samples from multiple international research groups (36,989 cases and 113,075 controls) performed by the Schizophrenia Working Group of the Psychiatric Genomics Consortium (2014) identified highly associated SNPs within MHC region. However, the existence of strong linkage disequilibrium (LD) across this gene-rich region (over 200 genes) makes it difficult to find the actual schizophrenia-associated variants (Rees et al. 2015).

Although HSP70 plays a wide range of functions in CNS, the association studies investigating the influence of HSP70 gene polymorphisms on the risk of developing, course, and psychopathology of schizophrenia are still strongly limited. The first three analyses have been performed on a Korean population exclusively (Pae et al. 2005; Kim et al. 2008; Pae et al. 2009). Previously, we explored the possibility that HSP70 gene polymorphisms might be involved in the susceptibility to paranoid schizophrenia and clinical presentation of the disease in Caucasian (Polish) individuals (Kowalczyk et al. 2014). The positive findings we obtained incline us to evaluation of more polymorphisms for associations with schizophrenia in larger cohorts.

In the present study, we performed a case–control analysis to investigate the potential association between the fourth variants of the *HSPA1B* gene (rs6457452, rs2763979, rs539689, and rs9281590) and the susceptibility to paranoid schizophrenia in a Caucasian Polish population. Taking into account the positive results of studies in the Korean population, we also investigated the impact of the *HSPA1B* polymorphisms on the clinical variables of the disease.

## Materials and Methods

### Subjects

The total sample set was composed of 377 unrelated patients: all were diagnosed with paranoid schizophrenia [153 (41%) females and 224 (59%) males; mean age ± SD 41.1 ± 12.3, range 18–74]. The patients had been recruited from the Department and Clinic of Psychiatry, Medical University of Silesia in Katowice and the Neuropsychiatric Hospital in Lubliniec. All of the patients were diagnosed as having paranoid type of schizophrenia according to DSM-IV-TR (Diagnostic and Statistical Manual of Mental Disorders, Fourth Edition, Text Revision). The final clinical diagnosis was assigned by two experienced independent psychiatrists based on the Structured Clinical Interview for DSM-IV Axis I Disorders, Clinical Version (SCID-I-CV, First et al. 1997). Exclusion criteria for patients were any other Axis I and Axis II diagnosis, neurological illness, endocrine disorders, and autoimmune diseases. All of the patients were hospitalized because of an acute or chronic schizophrenic psychosis. The severity of symptoms was measured by the Positive and Negative Syndrome Scale (PANSS, Kay et al. 1988) scale at the time of hospital admittance. Additional data were collected from medical records and through interviews with patients: age of onset (defined as the age at which the first psychotic symptoms appeared), presence or absence of suicide attempts, de novo/familial character of disease.

The control group was composed of 524 healthy, sex-frequency matched, unrelated individuals (volunteer blood donors of the Regional Centre of Blood Donation and Treatment in Katowice) [234 (45%) females and 290 (55%) males; mean age ± SD 39.8 ± 9.1, range 20–64]. Exclusion criteria for controls were current psychiatric problems, any other neurological disorders and family history of schizophrenia (verified by direct interview), chronic and acute physical illness such as infection, autoimmune, or allergic diseases.

All the participants were born in Poland and of Caucasian origin. All subjects had provided written consent prior to inclusion in the study. The Bioethics Committee of Medical University of Silesia approved the protocol of this study (No.KNW/0022/KB1/38/I/12).

### SNP Genotyping

Four SNPs (rs6457452, rs2763979, rs539689, and rs9281590) in the *HSPA1B* gene were selected as our candidate SNPs to explore the possible association with schizophrenia. Genomic DNA was extracted from whole blood samples using a QIAamp DNA Blood Mini Kit (Qiagen, Valencia, CA). The polymorphisms rs539689 and rs9281590 were genotyped by a PCR–restriction fragment length polymorphism (PCR–RFLP) assay. The regions spanning polymorphisms were amplified using the specific primers (Table 1) under previously described PCR conditions (Kowalczyk et al. 2014). Amplification was performed using a G-Storm GS1 thermal cycler (Gene Technologies LTD, Essex, UK) with a Taq polymerase (Epicentre, Biotechnologies) according to the manufacturer’s instructions in a reaction mix with a total volume of 25 μl. For RFLP detection, amplified PCR products were digested with the appropriate restriction enzymes (Thermo Fisher Scientific) and subsequently separated on 2–3% agarose gels stained with ethidium bromide. All enzymes and different-sized restriction fragments (allowing for discrimination between the two alleles) are summarized in Table 1.

**Table 1:** Summary of the PCR–RFLP genotyping method

SNP/location	Primers	Annealing (°C)	Enzyme: fragments in bp (allele)
rs539689 (G/C) Exon	F: 5′-CCTACGCCTTCAACATGAAG-3′ R: 5′-CAAAGTCCTTGAGTCCCAAC-3′	56	*Fnu*4HI: 356 (G)/230, 126 (C)
rs9281590 (AAGTT ins/del) 3′UTR	F: 5′-GTGGATTAGGGGCCTTTGTTCTTTAGT-3′ R: 5′-AGGGAACGAAACACCCTTACAGTATCA-3′	57	*Mse*I: 211, 33 (AAGTT del, A2)/129, 87, 33 (AAGTT ins, A1)

The polymorphisms rs6457452 and rs2763979 were genotyped using an allele-specific Taqman assay in a 96-well reaction plate. Each 25 μl reaction mixture contained 10 ng DNA, 12.5 μl TaqMan universal PCR Master Mix, and 1.25 μl primer/probe Mix (Life Technologies TaqMan SNP Genotyping Assay, ID: C_3052606_1 for rs2763979, and C_3052604_10 for rs6457452). The PCR and allelic discrimination were performed using the CFX96 real-time PCR detection system (Bio-Rad). The PCR thermal profile was as follows: initial denaturing at 95 °C for 10 min; 40 cycles of denaturation at 95 °C for 15 s; and annealing/extension at 60 °C for 1 min. Each 96-well plate contained 91 samples of an unknown genotype, two blank controls (reaction mixtures without the template), and three positive control samples for each genotype.

For quality control, approximately 5% of the randomly selected samples were repeated to validate the results of genotyping by PCR–RLP and TaqMan allelic discrimination assay.

### Statistical Analyses

Statistical analyses were performed using STATISTICA 10.0 PL (StatSoft, Cracow, Poland), StataSE 13.0 (StataCorp LP, TX, U.S.), and R software. Statistical significance was set at a *p* value below 0.05. All tests were two-tailed. Imputations were not done for missing data. Nominal and ordinal data were expressed as percentages, while interval data were expressed as mean value ± standard deviation. Distribution of variables was evaluated by the Shapiro–Wilk test and quantile–quantile (*Q*–*Q*) plot, and homogeneity of variances was assessed by the Levene test. Differences in the allele, genotype, and haplotype frequencies of the *HSPA1B* polymorphisms between groups were calculated using the *χ*^2^ test and the maximum likelihood *χ*^2^ test. The Hardy–Weinberg equilibrium at each polymorphism was examined based on the inbreeding coefficient using the Fischer’s exact test. The extent of the linkage disequilibrium (LD) expressed in terms of the *D*′ and *r*^2^ coefficients and haplotypes were estimated using the SNPStats. The odds ratio (OR) with a 95% confidence interval was used as the measure of the strength of the association between allele, genotype, and haplotype frequencies and paranoid schizophrenia occurrence. The associations between the genotypes, sex, and PANSS subscales and age of onset were calculated using either one-way or two-way ANOVA with Tukey’s post hoc test. Five inheritance models (co-dominant, dominant, recessive, over-dominant, and additive) were tested and the best fitted model was chosen according to the Akaike information criterion (AIC) and Bayesian information criterion (BIC).

## Results

There was a difference in age between males and females (38.5 ± 11.9 vs. 45.0 ± 11.8, *p* < 0.001) in the study group. There was no difference in age between males and females among the controls (40.0 ± 9.3 vs. 39.7 ± 8.8, *p* = 0.77). There was no difference in the percentage of females between study and control groups (40.6% vs. 44.7%; *χ*^2^ = 1.48, *p* = 0.22).

All of the polymorphisms were in Hardy–Weinberg equilibrium (HWE) both in the schizophrenia group [rs2763979 (*p* = 0.55), rs539689 (*p* = 0.45), rs9281590 (*p* = 0.37), rs6457452 (*p* = 0.60)] and in the controls [rs2763979 (*p* = 0.37), rs539689 (*p* = 0.09), rs9281590 (*p* = 0.53), rs6457452 (*p* = 0.13)].

### Single Marker Analyses

The distribution of *HSPA1B* polymorphisms in schizophrenic patients (*n* = 377) and controls (*n* = 524) is displayed in Table 2. There were no statistically significant differences in the genotype and allele frequencies between the schizophrenics and controls for all studied polymorphisms in the entire sample. However, for the rs539689 polymorphic site a strong tendency towards statistical significance (*p* = 0.051) in allele distribution between patients and controls was observed (rs539689C allele was more represented in controls, OR 0.83, 95% CI 0.68–1.00). To examine the gender-specific association with schizophrenia, we performed a sex stratified analysis, and the results are shown in Table 2. We found that rs539689 was significantly associated with schizophrenia in males, but not in females. The minor allele C had a protective effect in males [OR 0.73 (95% CI 0.61–0.88, *p* < 0.05)]. We also performed an analysis in the subgroups of patients with a positive (having at least one 1st or 2nd degree relative with schizophrenia) and negative family history of schizophrenia. There were 95 (25%) positive family history individuals. No associations were found in these subsamples.

**Table 2:** Analysis of genotype and allele distributions for the entire population and stratified by gender

SNP	Total group		*χ*^2^	*p*	Females		*χ*^2^	*p*	Males		*χ*^2^	*p*
	Patients	Controls			Patients	Controls			Patients	Controls		
Genotypes												
rs539689												
G/G	139 (36.9)	170 (32.4)	6.00	0.20	50 (32.7)	73 (31.2)	0.49	0.78	89 (39.7)	97 (33.5)	3.84	0.15
G/C	174 (46.1)	240 (45.8)			76 (49.7)	113 (48.3)			98 (43.8)	127 (43.8)		
C/C	64 (17.0)	114 (21.8)			27 (17.6)	48 (20.5)			37 (16.5)	66 (22.8)		
rs9281590												
A1/A1	45 (11.9)	63 (12.0)	0.19	0.91	22 (14.4)	25 (10.7)	1.55	0.46	23 (10.3)	38 (13.1)	0.97	0.62
A1/A2	159 (42.2)	228 (43.5)			62 (40.5)	106 (45.3)			97 (43.3)	122 (42.1)		
A2/A2	173 (45.9)	233 (44.5)			69 (45.1)	103 (44.0)			104 (46.4)	130 (44.8)		
rs2763979												
C/C	171 (45.4)	237 (45.2)	0.03	0.98	67 (43.8)	106 (45.3)	1.09	0.58	104 (46.4)	131 (45.2)	1.21	0.55
C/T	162 (43.0)	224 (42.8)			65 (42.5)	104 (44.4)			97 (43.3)	120 (41.4)		
T/T	44 (11.7)	63 (12.0)			21 (13.7)	24 (10.3)			23 (10.3)	39 (13.4)		
rs6457452												
C/C	314 (83.3)	445 (84.9)	–	0.46	122 (79.7)	198 (84.6)	–	0.12	192 (85.7)	247 (85.2)	–	0.08
C/T	61 (16.2)	73 (14.0)			29 (18.9)	36 (15.4)			32 (14.3)	37 (12.8)		
T/T	2 (0.5)	6 (1.1)			2 (1.3)	0			0	6 (2.0)		
Alleles												
rs539689												
G	452 (60.0)	580 (55.3)	3.80	0.051	176 (57.5)	259 (55.3)	0.36	0.55	276 (61.6)	321 (55.3)	4.07	**< 0.05**
C	302 (40.0)	468 (44.7)			130 (42.5)	209 (44.7)			172 (38.4)	259 (44.7)		
rs9281590												
A1	249 (33.0)	354 (33.8)	0.11	0.74	106 (34.6)	156 (33.3)	0.14	0.71	143 (31.9)	198 (34.0)	0.56	0.45
A2	505 (67.0)	694 (66.2)			200 (65.4)	312 (66.7)			305 (68.1)	382 (66.0)		
rs2763979												
C	504 (66.8)	698 (66.6)	0.01	0.92	199 (65.0)	316 (67.5)	0.51	0.47	305 (68.1)	382 (66.0)	0.56	0.45
T	250 (33.2)	350 (33.4)			107 (35.0)	152 (32.5)			143 (31.9)	198 (34.0)		
rs6457452												
C	689 (91.4)	963 (91.9)	0.15	0.70	273 (89.2)	432 (92.3)	2.18	0.14	416 (93.0)	531 (92.0)	0.59	0.44
T	65 (8.6)	85 (8.1)			33 (10.8)	36 (7.7)			32 (7.0)	49 (8.0)		

*N* (%); nominal associations are bolded

The estimated risks associated with *HSPA1B* polymorphisms were also tested according to different models of inheritance (co-dominant, dominant, recessive, over-dominant, log-additive). Unfortunately, we failed to find any evidence for a possible genetic contribution of the SNPs to schizophrenia susceptibility in either the entire sample or after stratification according to gender. However, we found that rs539689 showed a tendency to significant association with schizophrenia in the recessive model (comparing homozygous for the minor allele to the heterozygous and homozygous for the major allele) in the entire group [OR 0.74 (95% CI 0.52–1.03, *p* = 0.07)] and in males [OR 0.67 (95% CI 0.43–1.05, *p* = 0.08)], but not in females [OR 0.83 (95% CI 0.49–1.40, *p* = 0.48)], and C/C genotype was more represented in controls.

### Haplotype Analyses

The linkage disequilibrium (LD) analysis showed a weak LD between rs539689 and rs9281590 (*D*′ = 0.063, *p* < 0.05), and between rs539689 and rs2763979 (*D*′ = 0.094, *p* < 0.01), moderate LD between rs539689 and rs6457452 (*D*′ = 0.328, *p* < 0.01), and a strong linkage between rs9281590 and rs2763979 (*D*′ = 0.982, *p* < 0.001), rs9281590 and rs6457452 (*D*′ = 0.961, *p* < 0.001), and rs2763979 and rs6457452 (*D*′ = 0.947, *p* < 0.001).

The haplotype distribution of the four *HSPA1B* polymorphisms was not different between schizophrenia and control groups (Table 3), and schizophrenia predisposing haplotype was not identified. After the subjects were stratified by sex, the results were still non-significant.

**Table 3:** Haplotype analysis of the four *HSPA1B* SNPs (rs539689, rs9281590, rs2763979, rs6457452) in patients with paranoid schizophrenia and control subjects

Haplotype	Total group (*n* = 901)			Females (*n* = 387)			Males (*n* = 514)		
	Frequency	OR (95% CI)	*p*	Frequency	OR (95% CI)	*p*	Frequency	OR (95% CI)	*p*
G–A2–C–C	0.3944	1.00	–	0.3716	1.00	–	0.4083	1.00	–
C–A2–C–C	0.2656	0.89 (0.68–1.17)	0.40	0.2859	0.78 (0.51–1.18)	0.24	0.2536	1.00 (0.70–1.42)	0.98
G–A1–T–C	0.1453	1.06 (0.77–1.48)	0.71	0.1475	0.96 (0.57–1.63)	0.89	0.1445	1.16 (0.76–1.78)	0.49
C–A1–T–C	0.1039	0.75 (0.53–1.08)	0.12	0.0979	0.85 (0.47–1.51)	0.58	0.1073	0.71 (0.45–1.11)	0.14
C–A1–T–T	0.0484	0.84 (0.51–1.38)	0.50	0.0458	1.54 (0.69–3.43)	0.29	0.0480	0.56 (0.27–1.18)	0.13
G–A1–T–T	0.0314	1.19 (0.63–2.23)	0.59	0.0394	0.95 (0.40–2.25)	0.91	0.0280	1.56 (0.62–3.92)	0.34
Rare	0.0109	0.96 (0.39–2.38)	0.93	0.0117	1.52 (0.44–5.27)	0.51	0.0103	0.51 (0.12–2.13)	0.36

*OR* odds ratio, *CI* confidence interval

### Association of *HSPA1B* Variants with Clinical Parameters

Two-way ANOVA (sex × genotype) was used to study the individual effects of *HSPA1B* polymorphisms (rs539689, rs9281590, rs2763979) on the age of onset and the severity of symptoms measured by PANSS (Table 4). There was a significant main effect of rs539689 genotype for positive PANSS score (*p* < 0.001), general PANSS score (*p* < 0.01), and total PANSS score (*p* < 0.01). Post hoc analysis (Tukey’s tests) revealed that G/C and C/C carriers have lower mean scores of positive, general, and total PANSS, respectively, relative to genotype G/G [PANSS-P: G/C vs. G/G (*p* < 0.001), C/C vs. G/G (*p* < 0.05); PANSS-G: G/C vs. G/G (*p* < 0.01), C/C vs. G/G (*p* < 0.05); total PANSS: G/C vs. G/G (*p* < 0.01), C/C vs. G/G (*p* < 0.05)]. Two-way ANOVA also indicated a statistical trend for a main effect of rs539689 genotype on negative PANSS score (*p* = 0.086). Furthermore, there was a significant interaction between sex and rs9281590 genotype for general PANSS score. A Tuckey’s post hoc test showed significant differences between males and females who carried the A1/A1 genotype (*p* < 0.01). There were also trends towards significance for an interaction between sex and rs9281590 genotype for total PANSS score (*p* = 0.065), and between sex and rs2763979 genotype for general PANSS score (*p* = 0.089).

**Table 4:** Results from the two-way ANOVA (sex, genotype) on Positive and Negative Syndrome Scale (PANSS) and age of onset

SNPs	Clinical variables	Females			Males			*p*
		G/G	G/C	C/C	G/G	G/C	C/C	
rs539689	PANSS-P	22.6 ± 5.8	20.2 ± 5.4	19.3 ± 5.3	23.3 ± 6.2	20.4 ± 5.9	21.8 ± 5.6	0.097^a^/**< 0.001**^b^**/**0.428^c^
	PANSS-N	25.2 ± 5.9	24.3 ± 6.6	23.0 ± 5.7	25.9 ± 6.5	25.4 ± 6.4	23.8 ± 5.0	0.209^a^/**0.086**^b^**/**0.961^c^
	PANSS-G	43.8 ± 8.2	41.2 ± 8.7	40.7 ± 6.3	46.8 ± 9.6	42.6 ± 8.9	42.1 ± 7.9	0.097^a^/**< 0.01**^b^**/**0.730^c^
	Total PANSS	91.8 ± 17.1	85.7 ± 17.8	83.0 ± 13.2	96.0 ± 18.8	88.5 ± 17.0	87.7 ± 15.8	**< 0.05**^a^/**< 0.01**^b^**/**0.907^c^
	Age of onset	28.4 ± 8.2	27.4 ± 7.2	28.3 ± 7.1	23.4 ± 5.4	24.3 ± 5.9	25.3 ± 6.1	**< 0.001**^a^/0.604^b^**/**0.394^c^

		A1/A1	A1/A2	A2/A2	A1/A1	A1/A2	A2/A2	
rs9281590	PANSS-P	19.8 ± 6.1	21.9 ± 5.6	20.2 ± 5.4	21.0 ± 5.6	21.7 ± 6.0	22.0 ± 6.2	0.195^a^/0.299^b^**/**0.300^c^
	PANSS-N	22.5 ± 5.8	25.1 ± 6.3	24.2 ± 6.2	26.3 ± 6.4	24.9 ± 6.1	25.5 ± 6.4	**< 0.05**^a^/0.828^b^**/**0.153^c^
	PANSS-G	38.5 ± 6.1	43.5 ± 7.8	41.7 ± 8.8	45.9 ± 9.8	43.4 ± 8.6	44.5 ± 9.7	**< 0.01**^a^/0.704^b^**/< 0.05**^c^
	Total PANSS	80.8 ± 15.1	90.5 ± 16.9	86.2 ± 17.3	93.2 ± 18.8	90.1 ± 16.9	92.1 ± 18.7	**< 0.01**^a^/0.520^b^**/0.065**^c^
	Age of onset	26.1 ± 7.4	27.0 ± 8.1	29.4 ± 6.8	24.7 ± 6.1	23.8 ± 5.7	24.3 ± 5.8	**< 0.001**^a^/0.101^b^**/**0.181^c^

		C/C	C/T	T/T	C/C	C/T	T/T	
rs2763979	PANSS-P	20.3 ± 5.4	21.6 ± 5.7	20.0 ± 6.1	22.0 ± 6.2	21.7 ± 6.0	21.0 ± 5.6	0.209^a^/0.468^b^**/**0.480^c^
	PANSS-N	24.3 ± 6.1	25.0 ± 6.3	22.5 ± 6.0	25.5 ± 6.4	24.9 ± 6.1	26.3 ± 6.4	**< 0.05**^a^/0.864^b^**/**0.187^c^
	PANSS-G	42.1 ± 8.7	42.9 ± 8.1	38.7 ± 6.2	44.5 ± 9.7	43.4 ± 8.6	45.9 ± 9.8	**< 0.01**^a^/0.787^b^**/0.089**^c^
	Total PANSS	86.8 ± 17.2	89.5 ± 17.2	81.2 ± 15.3	92.1 ± 18.7	90.1 ± 16.9	93.2 ± 18.8	**< 0.01**^a^/0.680^b^**/**0.141^c^
	Age of onset	29.2 ± 7.0	27.1 ± 7.9	26.4 ± 7.4	24.4 ± 5.8	23.7 ± 5.7	24.7 ± 6.1	**< 0.001**^a^/0.144^b^**/**0.286^c^

Values represent mean PANSS scores/mean age of onset (years) ± standard deviation

Nominally significant *p* values are given in bold

*PANSS-P* positive scale, *PANSS-N* negative scale, *PANSS-G* general psychopathology scale

^a^Main effect of sex

^b^Main effect of genotype

^c^Sex × genotype interaction

Main effect of rs6457452 genotype on clinical parameters was assessed with one-way ANOVA (T/T genotype carrier group was too small to perform a two-way ANOVA with genotype and sex as factors). There was no significant rs6457452 genotype effect for PANSS and age of onset.

### Association of *HSPA1B* Variants with Suicidal Behavior

We further examined the potential association between suicidal behavior and individual polymorphisms in the entire sample and after stratification according to gender based on different genetic models. Out of the four SNPs tested, only rs539689 showed significant association to suicide attempt (Table 5). Two the best fits inheritance models with the lowest BIC were co-dominant and dominant either among entire patient population or among patients stratified by gender. In the entire group, the analysis revealed that C/C and G/C genotypes of the *HSPA1B* rs539689 were protective against suicidal behavior under dominant and co-dominant models. Similarly, in males, those two genotypes were found to be associated with decreased risk of suicide attempts. In females, the genotype C/C and C/C–G/C appeared to have a reduced risk for suicide in co-dominant model and dominant models, respectively, but the associations were weaker than in males.

**Table 5:** Analysis of different inheritance models for the SNP rs539689 between suicidal attempters (SA) and no-suicidal attempters (NSA) with a paranoid schizophrenia diagnosis

Model	Genotype	Total group (*n* = 377)				Females (*n* = 153)				Males (*n* = 224)			BIC
		SA/NSA N (%)	OR (95% CI)	*p*	BIC	SA/NSA *N* (%)	OR (95% CI)	*p*	BIC	SA/NSA *N* (%)	OR (95% CI)	*p*	
Co-dominant	G/G G/C C/C	43(64.2)/96(31.0) 19(28.4)/155(50.0) 5(7.5)/59(19.0)	1.00 **0.27 (0.15–0.50)** **0.19 (0.07–0.50)**	**< 0.001**	344.9	12(52.2)/38(29.2) 10(43.5)/66(50.8) 1(4.3)/26(20.0)	1.00 0.48 (0.19–1.22) **0.12 (0.01–0.99)**	**< 0.05**	137.9	31(70.5)/58(32.2) 9(20.4)/89(49.4) 4(9.1)/33(18.4)	1.00 **0.19 (0.08–0.43)** **0.23 (0.07–0.70)**	**< 0.001**	216.8
Dominant	G/G G/C–C/C	43(64.2)/96(31.0) 24(35.8)/214(69.0)	1.00 **0.25 (0.14–0.44)**	**< 0.001**	339.4	12(52.2)/38(29.2) 11(47.8)/92(70.8)	1.00 **0.38 (0.15–0.93)**	**< 0.05**	135.2	31(70.5)/58(32.2) 13(29.5)/122(67.8)	1.00 **0.20 (0.10–0.41)**	**< 0.001**	211.4
Recessive	G/G–G/C C/C	62(92.5)/251(81.0) 5(7.5)/59(19.0)	1.00 **0.34 (0.13–0.89)**	**< 0.05**	358.5	22(95.7)/104(80.0) 1(4.3)/26(20.0)	1.00 0.18 (0.02–1.41)	0.08	135.3	40(90.9)/147(81.6) 4(9.1)/33(18.4)	1.00 0.45 (0.15–1.33)	0.12	230.3
Over-dominant	G/G–C/C G/C	48(71.6)/155(50.0) 19(28.4)/155(50.0)	1.00 **0.40 (0.22–0.70)**	**< 0.001**	353.9	13(56.5)/64(49.2) 10(43.5)/66(50.8)	1.00 0.75 (0.31–1.82)	0.52	139.2	35(79.6)/91(50.6) 9(20.4)/89(49.4)	1.00 **0.26 (0.12–0.58)**	**< 0.001**	219.8
Log-additive	–	–	**0.35 (0.23–0.55)**	**< 0.001**	340.6	–	**0.41 (0.20–0.85)**	**< 0.05**	133.2	–	**0.33 (0.19–0.59)**	**< 0.001**	215.5

Nominally significant *p* values are given in bold

*OR* odds ratio, *CI* confidence interval, *BIC* Bayesian information criterion

We also performed allele association analyses. The minor allele C of rs539689 was underrepresented among patients with records of suicidal behavior [OR 0.35 (95% CI 0.22–0.54), *p* < 0.001]. After the subjects were stratified by sex, the association was clearly stronger in males [OR 0.32 (95% CI 0.18–0.56), *p* < 0.001] than in females [OR 0.42 (95% CI 0.21–0.86), *p* < 0.05]. Haplotype association analysis with respect to suicidal attempt was performed as well. The C–A2–C–C haplotype was found to exert a significant protective effect in comparison with the most frequent G–A2–C–C haplotype in the entire sample [OR 0.26 (95% CI 0.13–0.51, *p* < 0.001)], in males [OR 0.24 (95% CI 0.10–0.58, *p* < 0.01)], and marginally in females [OR 0.29 (95% CI 0.09–0.98, *p* < 0.05)].

## Discussion

In our previous work (Kowalczyk et al. 2014), we explored the possibility that *HSPA1A* (+190G/C), *HSPA1B* (+1267A/G), and *HSPA1L* (+2437T/C) gene polymorphisms might be involved in the susceptibility to paranoid schizophrenia and clinical presentation of the disease in the Polish population. To the best of our knowledge, study performed in our department was the first investigating the association between *HSP70* gene polymorphisms and schizophrenia in Caucasian individuals. The obtained results provided the first evidence that the *HSPA1A* + 190CC genotype and + 190C allele may potentially confer increased risk for paranoid schizophrenia in Caucasian Polish residents in a sex-dependent manner. We also identified C–G–T haplotype as predisposing to schizophrenia, and demonstrated the effects of *HSPA1A* and *HSPA1B* genotypes on psychopathology and age of onset.

In this paper, we evaluated the association between the other four variants of the *HSPA1B* gene (rs6457452, rs2763979, rs539689, and rs9281590) and the susceptibility to paranoid schizophrenia. Our patient group was larger than those in previous study (samples of previous study were included), but still homogenous with respect to ethnicity, geographic region, and schizophrenia subtype. One of the main problems of association studies in schizophrenia is non-replication and inconsistency between studies. Such discrepancy among studies may reflect differences across populations, but also may be due to heterogeneity of samples (e.g., mixed paranoid schizophrenia with other subtypes). Additionally, all analyses, we performed, were stratified according to gender. It is widely accepted that schizophrenia is a sexually dimorphic disease, and gender differences have been extensively described, especially with respect to age of onset, course of illness, clinical symptomatology, and treatment response (Grossman et al. 2006). In addition, a number of sex-specific genetic associations with schizophrenia risk have been reported for several genes such as *ZNF804A* (Zhang et al. 2011), the myelin transcription factor 1-like (*MYT1L*) (Li et al. 2012), and interferon γ (*IFN*-*γ*) (Paul-Samojedny et al. 2011).

The two selected SNPs may influence the expression of the *HSPA1B* gene. The *HSPA1B* rs2763979 (located outside the minimal promoter region) was found to play a role in modulating HSP70-1b expression, and the rs2763979T allele was sufficient to reduce expression of transcriptional reporter constructs, when compared with the rs2763979C allele (Guo et al. 2011). The minor allele of rs9281590 was found to reduce *HSPA1B* mRNA stability. *HSPA1B* expression was also lower in homozygote individuals for the minor rs9281590 allele, as compared with homozygous subjects for the major allele, thus reinforcing a role of this variant on gene expression (Marucci et al. 2010). Taking into account that HSP70-1b together with HSP70-1a are the major stress-inducible family members, we hypothesized that decreased synthesis of HSP70-1b protein may impair the cellular response to stress and be one of the predisposing factors to schizophrenia.

It should be emphasized that our study is the first analyzing the association between rs2763979, rs9281590, and rs6457452 SNPs of *HSPA1B* and schizophrenia. Unfortunately, we found no association between these three polymorphisms and paranoid schizophrenia itself, but rs9281590 had some impact on clinical symptoms, as significant interaction between sex and rs9281590 genotype for general PANSS score was observed. Males who carried the A1/A1 genotype had higher mean scores of general PANSS than females with the same genotype (45.9 vs. 38.5). Similar differences have been revealed for total PANSS score (93.2 males vs. 80.8 females), but did not achieve statistical significance (*p* = 0.065). Growing evidence indicates that the antioxidant defense system failure may be associated with both pathophysiology and psychopathology of schizophrenia, especially as the use of antioxidants has been found to improve some of the psychopathological symptoms of schizophrenia (Bitanihirwe and Woo 2011; Calabrese et al. 2017). Oxidative stress results from the imbalance of reactive oxygen (ROS) and nitrogen species (RNS), which are generated from various environmental exposures, but also from the metabolism neurotransmitters such as dopamine and glutamate. The failure of antioxidant defense system to neutralize free-radicals may result in tissue damage, and may have an impact on neurotransmission and, ultimately, clinical symptoms of schizophrenia (Flatow et al. 2013). Clinical studies have shown that oxidative stress is associated with the PANSS subscales scores, and with cognitive deficits in schizophrenia (Huang and Liu 2017). HSP70 has been reported to be part of the antioxidant defense system, which includes enzymatic antioxidants such as superoxide dismutase (SOD), glutathione peroxidase (GPx), or glutathione reductase (GR), and non-enzymatic antioxidants. It has been postulated that both oxidative stress and antioxidants may impact on HSP70 expression, and decrease in HSP70 expression may lead to the increasing in ROS generation (Guo et al. 2007; Calabrese et al. 2010). It was also found that HSP70 can regulate cellular redox status through increasing the activities of antioxidant enzymes GPx and GR in cells exposed to hypoxia/ischemia, which may be important in HSP70 cytoprotective mechanism (Guo et al. 2007). Zhang et al. (2006) have noted a significant difference in antioxidant enzyme activity between schizophrenic males and females suggesting that female patients may have suffered from more oxidative damage. Currently, despite the presence the same A1/A1 genotype, associated with lower expression of *HSPA1B*, females had a milder clinical phenotype than males, which may be attributed to the neuromodulatory and neuroprotective effect of oestrogen, a hormone that can regulate HSP70 expression in neuronal cells (Hou et al. 2010). Previously, an association between the presence of the *HSPA1B* rs9281590 A2 allele and overexpression of non-cognitive abnormalities within a population affected by Alzheimer’s disease has been reported (Clarimon et al. 2003).

On the contrary, a strong tendency towards statistical significance (*p* = 0.051) in the allelic distribution of the *HSPA1B* rs539689 polymorphism between patients and controls was observed in this report, and the rs539689G allele was more represented in patients with paranoid schizophrenia. After gender stratification, we found rs539689 significantly associated with schizophrenia in males (the minor allele C exerted a protective effect against schizophrenia), but not in females. It could be argued that females with the risk allele of rs539689 would not develop schizophrenia because of high level of circulating oestrogen. Oestrogen is known to have diverse neuroprotective properties (e.g., enhance neurogenesis, synaptic plasticity, and connectivity). It can also modulate dopamine, serotonin, and glutamate neurotransmission in the brain (Hayes et al. 2012). Several studies indicated that oestrogen-mediated neuroprotective effect against stress-induced brain damage may be attributed to up-regulation of heat shock protein expression (Hou et al. 2010). So far, except for our research, rs539689 polymorphism was analyzed in schizophrenia in the Korean population exclusively (Kim et al. 2008; Pae et al. 2009). In the first study, Kim et al. (2008) showed no impact of *HSPA1B* rs539689 polymorphism on the susceptibility to schizophrenia. However, they found A–C–C–**G** haplotype associated with a significantly increased risk of developing schizophrenia, and a significant protective effect exerted by the G–G–G–**C** haplotype. We failed to find any schizophrenia predisposing haplotype, but in our study rs539689C allele was protective against schizophrenia and in report published by Kim and colleagues rs539689C allele was a part of a protective haplotype. More recently, Pae et al. (2009) have studied the influence of the 5 SNPs of HSP70 genes on clinical presentation and drug response in schizophrenic patients. They found rs539689 SNP in *HSPA1B* gene marginally associated with PANSS positive scores at discharge, and two haplotypes significantly associated with the difference in PANSS total and negative subscales from baseline to discharge. The authors speculated that HSP mutations may differentially influence negative and positive phenotypes in accordance with the molecular role of these proteins. Current study also showed an impact of *HSPA1B* rs539689 SNP on the clinical presentation of schizophrenia. Carriers of genotype G/G have significantly higher mean scores of positive, general, and total PANSS than heterozygous and homozygous C/C patients. We demonstrated the effects of genotypes of *HSPA1B* + 1267A/G polymorphism on schizophrenia psychopathology (positive PANSS scores) in our previous study (Kowalczyk et al. 2014). A significant association of the *HSPA1B* + 1267A allele with schizophrenia development (but no psychopathology) in Koreans has also been reported (Pae et al. 2005). These results suggest that *HSPA1B* polymorphisms may differentially contribute to the development of schizophrenia and to the clinical manifestation of the disease across various human populations.

Another important finding of the current study was a strong significant association between the *HSPA1B* rs539689 polymorphism and attempted suicide in patients with schizophrenia. The mechanism behind this association is unclear (there is no direct evidence of the functional effects of this SNP). However, we can predict that rs539689 is in LD with another functional SNP which may have effect on gene expression (we discuss this point in detail later in text). The genotype analysis revealed that C/C genotype of the *HSPA1B* rs539689 was protective against suicidal behavior in entire sample (OR 0.19), *p* < 0.001), in males (OR 0.23, *p* < 001), and in females (OR 0.12, *p* < 0.05). In allele association analyses, the odds ratio was 0.35 (*p* < 0.001, entire sample), 0.32 (*p* < 0.001, males), and 0.42 (*p* < 0.05, females), indicating that the presence of the minor allele C of rs539689 protects against the suicidal behavior. Finally, in haplotype analysis the C–A2–C–C haplotype was shown to exert a significant protective effect against suicide in the entire sample (OR 0.26, *p* < 0.001), in males (OR 0.24, *p* < 0.01), and marginally in females (OR 0.29, p < 0.05). Note that in females all associations were weaker than in males, but there is a possibility of type I error caused by small sample sizes available for analysis after stratification according to gender. Unfortunately, there are no studies reporting the relationships between polymorphisms in HSP genes and suicidal behavior. Family and twin studies indicate that genetic factors contribute as much as 30–50% to suicidal behavior. As yet, genes involved in the regulation of serotonergic, dopaminergic, GABA-ergic systems, hypothalamic–pituitary–adrenal (HPA) axis, and brain-derived neurotrophic factor have been the most extensively examined in suicide, with no consistent associations recorded (Ganança et al. 2016). Kim et al. (2007) explored suicide candidate genes associated with schizophrenia, and found 70 differentially expressed genes possibly involved in suicide. Among these genes, only *HSPB1* (down-regulated) belongs to the HSP family. However, two genes map to the MHC locus (*HLA*-*DPA* and *HLA*-*DRA*, up-regulated).

The *HSPA1B* gene is located in the MHC class III region on chromosome 6p21.3 (Milner and Campbell 1990). One of the hallmarks of the MHC region is high gene density and high and long-range linkage disequilibrium (LD) between loci and SNP markers across the MHC. The four population-based studies have found that 34–44% of the HLA alleles are in strong correlation with one or more SNPs, also located at a considerable distance from the HLA allele (de Bakker et al. 2006). Therefore, it should be considered that a statistical significance found between rs539689 polymorphic site and schizophrenia/suicidal behavior may be due to LD with the tumor necrosis factor-α (*TNF*-*α*) SNPs or other adjacent genes (e.g., HLA genes), since TNF gene cluster and HSP70 gene families are at a distance of 600 kb from each other. Pae et al. (2009) have not observed LD between rs539689 and another *HSPA1B* SNP rs1061581, whereas Schroeder et al. (1999) noted the LD between *TNF* locus and *HSPA1B*. In our study, rs539689 was only in a weak LD both with rs9281590 and rs2763979 SNPs, and in a moderate LD with rs6457452. Numerous studies have demonstrated association between HLA alleles (HLA-A9, HLA-A10, HLA-DRB1, HLA-DQB1) and schizophrenia susceptibility. *NOTCH4*, another gene that maps to the HLA locus, has been implicated as a schizophrenia risk gene in various studies (Mokhtari and Lachman 2016). Positive association has also been found between TNF-α-G308A functional polymorphism and schizophrenia (Czerski et al. 2008; Sacchetti et al. 2007). Interestingly, Sacchetti et al. (2007) observed a gender-dependent association between TNF-α and schizophrenia. Similarly to our results (concerning rs539689), they found that A allele of the -G308A polymorphism in TNF-α gene increased the susceptibility for schizophrenia only in males. Additionally TNF-α-308G/G genotype has been increased in males with suicide behavior versus control group (Omrani et al. 2009), but schizophrenic patients were not included into this study.

To conclude, our case–control study provided evidence that variants in *HSPA1B* gene contribute to schizophrenia susceptibility, psychopathology, and suicidal behavior in schizophrenic patients, which is a novel finding. Further studies with larger sample size and in different ethnic backgrounds are needed to clarify the role of *HSPA1B* in the pathogenesis of schizophrenia.
